# Acute pancreatitis after major spine surgery: a case report and literature review

**DOI:** 10.1186/s13013-018-0170-2

**Published:** 2018-11-08

**Authors:** Daniela Ghisi, Alessandro Ricci, Sandra Giannone, Tiziana Greggi, Stefano Bonarelli

**Affiliations:** 1 Anesthesia, Intensive Care and Pain Therapy, Istituto Ortopedico Rizzoli, via G. C. Pupilli 1, 40136 Bologna, Italy; 2 Department of Spinal Deformity Surgery, Istituto Ortopedico Rizzoli, via G. C. Pupilli 1, 40136 Bologna, Italy

**Keywords:** Pancreatitis, Scoliosis, Prone position, Amylase, Lipase, Postoperative complications

## Abstract

**Background:**

Acute pancreatitis has been described as potential complication of both abdominal and non-abdominal surgeries. The pathogenetic mechanism underlying acute pancreatitis in spine surgery may include intraoperative hemodynamic instability causing prolonged splanchnic hypoperfusion, as well as mechanical compression of the pancreas due to scoliosis correction, with a higher risk in cases of more extended fusions, especially in young adults with lower body mass index (BMI).

**Case presentation:**

We report here a case of postoperative acute pancreatitis with benign evolution in a young female patient after the first and second surgery of a two-stage correction of right thoracic idiopathic scoliosis.

In December 2017, the patient underwent first-stage T4-L3 posterior arthrodesis with T7-T12 osteotomies and temporary magnetic bar. Intraoperative blood loss required massive transfusion. In the immediate postoperative period, the patient started reporting nausea/vomiting, abdominal pain at pressure, moderate meteorism, abdominal distension, hypoactive bowel sounds, and fever. Laboratory tests indicated a progressive increase in aspartate aminotransferase, alanine aminotransferase, serum amylase, lipase, phospho-creatine kinase, and reactive C-protein. A CT scan showed free abundant abdominal fluid in the hepatic, renal, pancreatic, and pelvic regions. After the diagnosis, a hypolipidic diet was initiated, and good hydration per os was maintained. After gastroenterologic consultation, somatostatin, rifaximin, and ursodehoxycholic acid were initiated and maintained for 8 days. In the following days, laboratory tests showed a slow but consistent decrease in liver and pancreatic enzymes until normalization. In January 2018, the patient underwent second-stage surgery with removal of magnetic bar, definitive posterior fusion, and instrumentation T4-L3. Laboratory tests showed a second, even more significant, increase in the amylase and lipase level and a moderate increase in the reactive C-protein. Therapy was maintained until complete normalization of amylase and lipase levels.

**Conclusions:**

Early recognition of symptoms plays a key role in preventing severe morbidity after scoliosis surgery. When symptoms suggest abdominal complication, pancreatic and liver enzymes are to be evaluated for posing prompt diagnosis. Gastroenterologic consultation and eventual imaging are further steps in differential diagnosis and treatment of this rare complication.

**Electronic supplementary material:**

The online version of this article (10.1186/s13013-018-0170-2) contains supplementary material, which is available to authorized users.

## Background

Acute pancreatitis has been described in literature as a potential complication of both abdominal and non-abdominal surgeries. A recent prospective study reported an incidence of 7.4% in 176 young patients after surgery for scoliosis correction [1].

Although the incidence of this potential postoperative complication of spine surgery has decreased since 1991 [1–3], acute pancreatitis needs to be considered in patients showing at least two of the following criteria: (1) abdominal pain or nausea/vomiting, (2) at least threefold increase in serum lipase level compared with the upper limit of normal level, (3) characteristic findings of acute pancreatitis on transabdominal ultrasonography or computed tomography (CT) [4]. Patients with neuro-fibromatosis type 1, Marfan syndrome, and cerebral palsy are at higher risk [1].

The pathogenetic mechanisms underlying acute pancreatitis in spine surgery may include intraoperative hemodynamic instability causing prolonged splanchnic hypoperfusion [1, 3] as well as mechanical compression of the pancreas due to scoliosis correction [1], with a higher risk in cases of more extended fusions, especially in young adults with lower body mass index (BMI) [5].

A two-stage technique for posterior arthrodesis has been introduced in severe scoliosis and advocated for reduction in perioperative complications [6].

We report here a case of postoperative acute pancreatitis in a young female patient after the first and second surgery of a two-stage correction of right thoracic idiopathic scoliosis.

## Case presentation

**Fig. 1:**
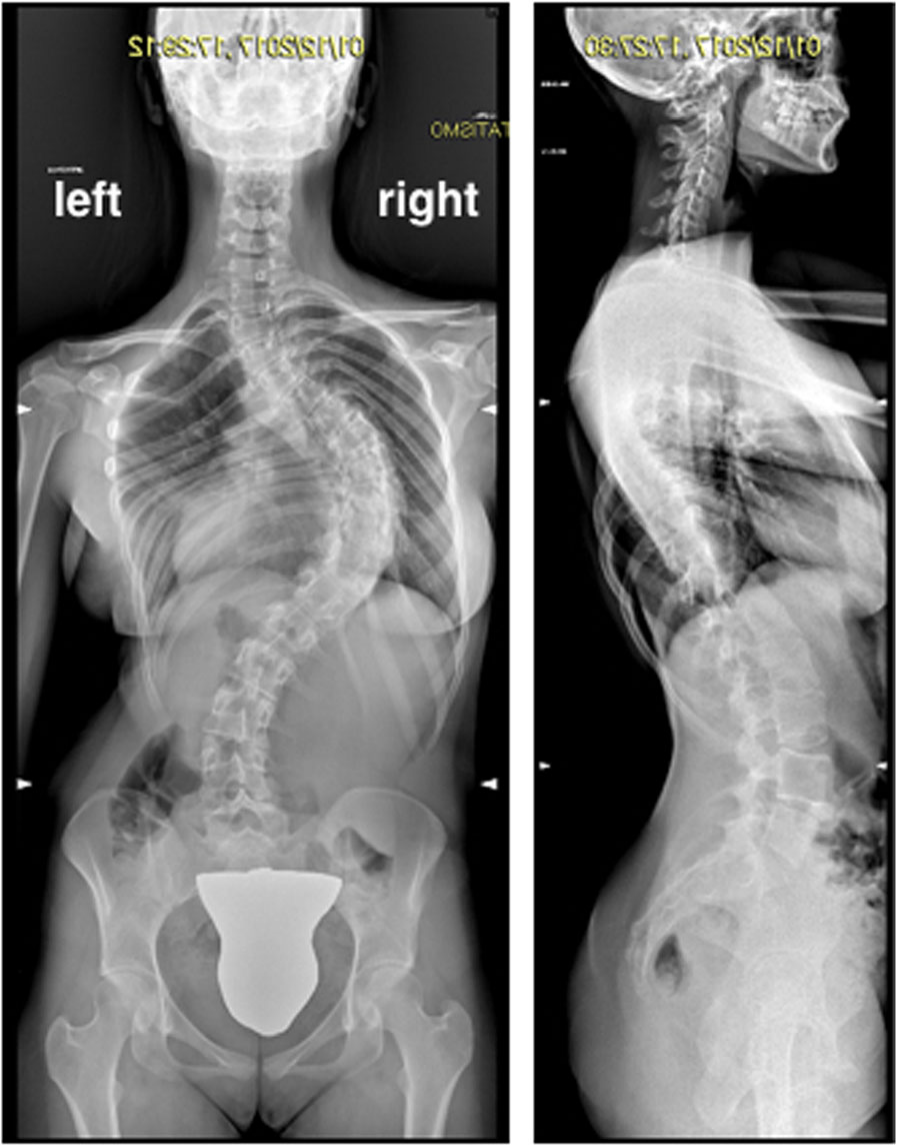
X-rays of the patient before surgical correction of her thoracic right scoliosis of 95°, thoracic kyphosis of 70°, and lumbar lordosis of 62°

Between the 1st and the 23rd of December 2017, a 15-year-female patient (weight 66 kg, height 174 cm, body mass index (BMI) 21.85 kg/m^2^) was hospitalized in our institution for severe idiopathic scoliosis (thoracic right scoliosis of 95°, thoracic kyphosis for 70°, lumbar lordosis of 62°, Risser grade 3 for ossification of the iliac crest apophysis (Fig. 1). An additional image file shows a 3D CT scan of the column (see Additional file 1). The 70° kyphosis is measured on column X-rays (see Additional file 2) with indication for a two-stage posterior arthrodesis including first-stage instrumentation with growing magnetic rod and second-stage posterior fixation. Parents signed consent for clinical data management for scientific purposes.

Patient’s clinical history was significant only for allergic rhinitis due to environmental allergens.

**Fig. 2:**
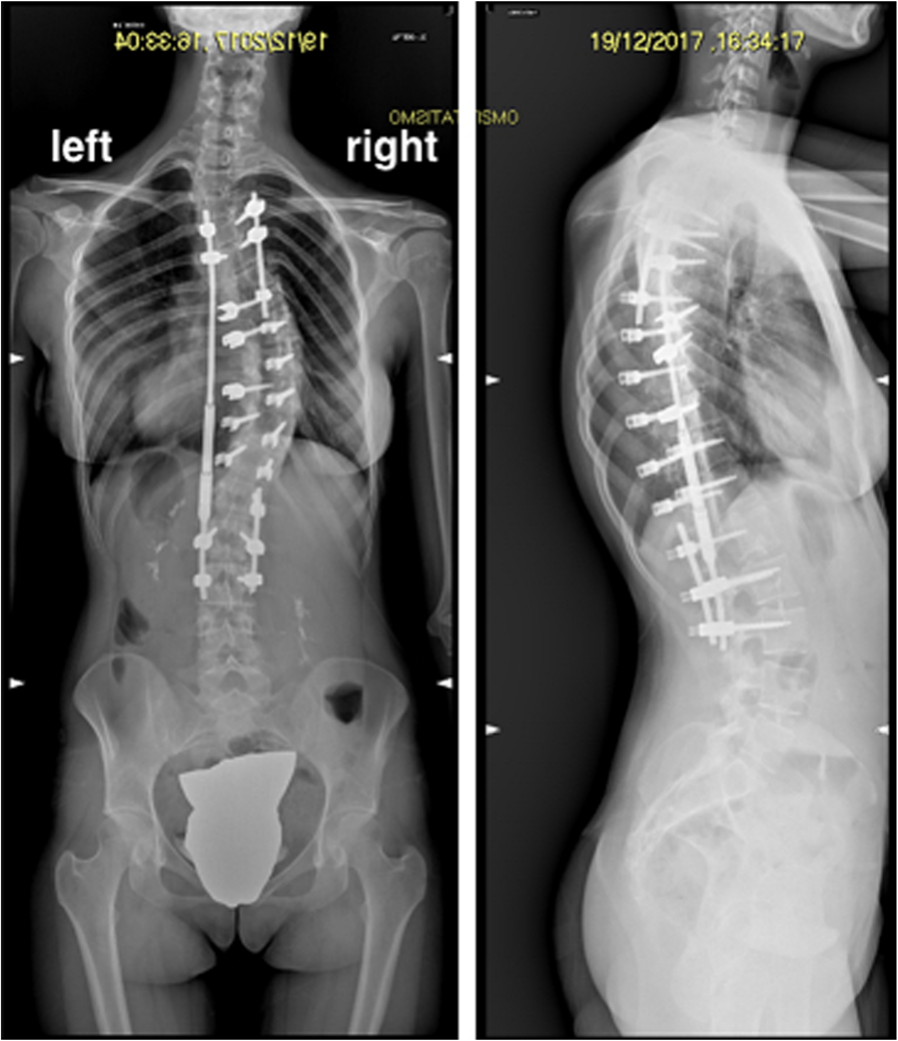
X-rays after first-stage T4-L3 posterior arthrodesis with T7-T12 osteotomies and temporary magnetic bar

**Fig. 3:**
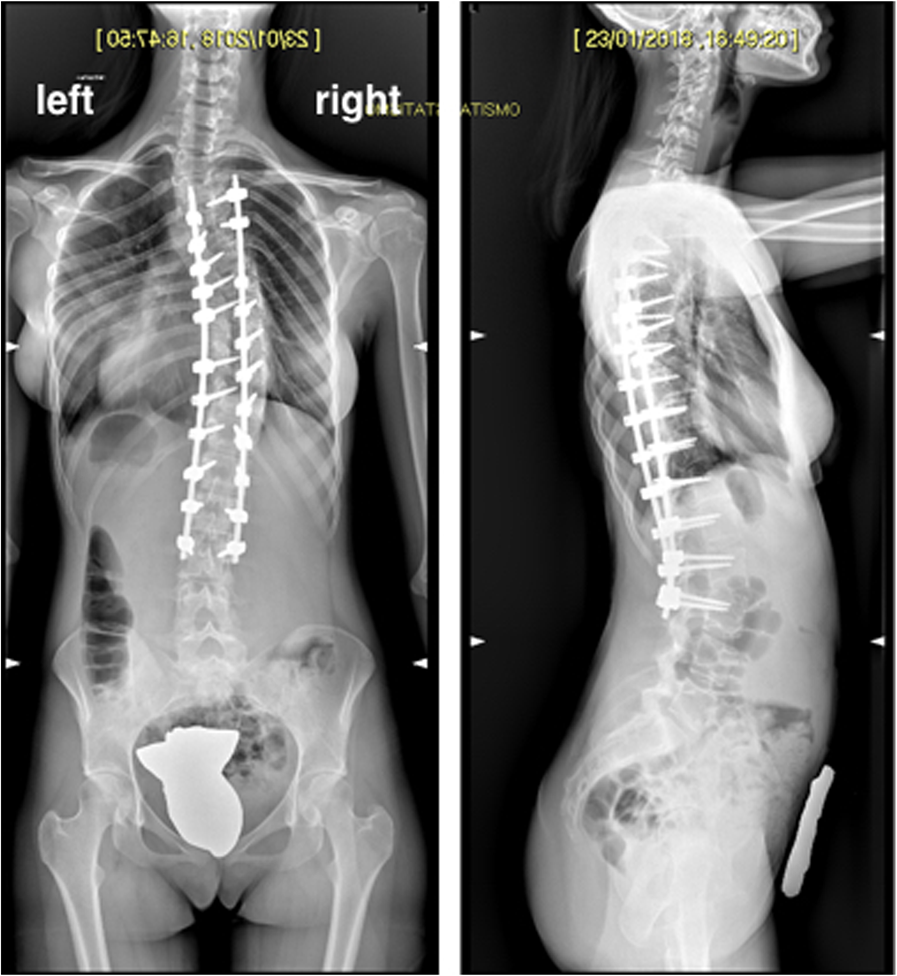
X-rays after second stage: removal of magnetic bar and definitive posterior fusion and instrumentation T4-L3

On the 4th of December, the patient underwent first-stage T4-L3 posterior arthrodesis with T7-T12 osteotomies and temporary magnetic bar (Figs. 2 and 3). Intraoperative blood loss required transfusion with 630 ml of autologous blood from cell saver, three units of homologous blood, and 600 ml of fresh frozen plasma. Surgery went otherwise uneventfully, and patient was transferred in spontaneous breathing to the postoperative intensive care unit after awakening from general anesthesia for postoperative monitoring.

The day after surgery, the patient started complaining of nausea and mild abdominal pain during pressure. One episode of vomiting occurred on postoperative day 1. Moderate tympanites, abdominal distension, and hypoactive bowel sounds were also noted. Laboratory tests indicated an increase in aspartate aminotransferase (AST, 235 U/L) and alanine aminotransferase (ALT, 152 U/L) levels as well as elevated serum amylase (341 U/L), lipase (704 U/L), phospho-creatine kinase (CK, 2313 U/L), and reactive C-protein (RCP, 16.73 mg/dL).

A first ultrasound scan of the abdomen was performed showing significant meteoric intestinal distension. The pancreatic region was therefore not explorable.

During the second postoperative day, patient complained of more severe pain during pressure in the hypocondrium and mesogastrium. Bowel was open to gas. A CT scan was performed, showing free abundant abdominal fluid in the hepatic, renal, pancreatic, and pelvic regions. Temperature raised to 38 °C and RCP increased to 18.49 mg/dL. Liver and pancreatic enzymes decreased mildly (AST = 165 U/L, ALT = 135 U/L, amylase = 261 U/L, lipase = 319 U/L, CK = 1827 U/L).

In the following days, laboratory tests showed a slow but consistent decrease in liver and pancreatic enzymes and the second CT scan performed on the 6th postoperative day demonstrated only moderate Douglas pouch effusion. After the diagnosis, a hypolipidic diet was initiated; good hydration per os was maintained; and somatostatin 0.1 mg three times a day per os, rifaximin 400 mg twice a day per os, ursodehoxycholic acid 300 mg twice a day per os were suggested for 8 days by the gastroenterologist who evaluated the patient, posed the diagnosis of acute pancreatitis, and suggested second-stage surgery to be delayed.

Patient was then transferred to the floor and discharged home 19 days after surgery.

On the 15th of January, after complete normalization of the laboratory tests and preoperative gastroenterologic evaluation, the patient underwent second-stage surgery with removal of magnetic bar, definitive posterior fusion, and instrumentation T4-L3. After surgery, the patient was transferred to the intensive care unit.

**Fig. 4:**
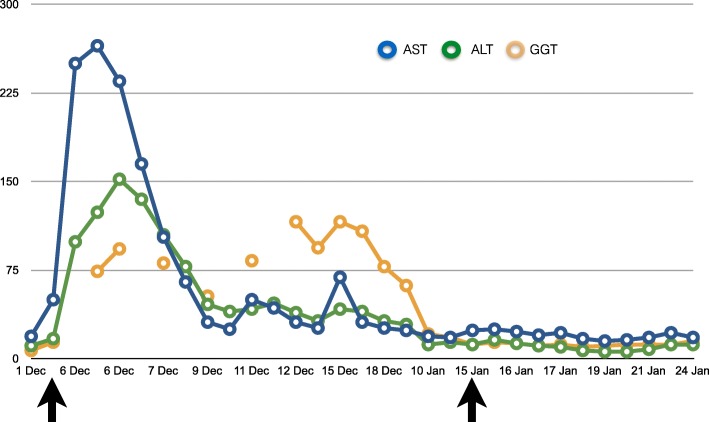
Trend of hepatic enzymes (AST, ALT, GGT) before and after first- and second-stage surgeries (arrows)

**Fig. 5:**
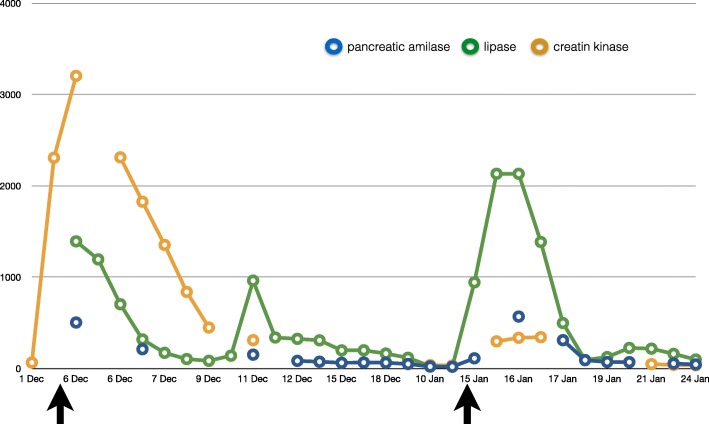
Trend of pancreatic enzymes (amylase and lipase) and creatine kinase before and after first- and second-stage surgeries (arrows)

**Fig. 6:**
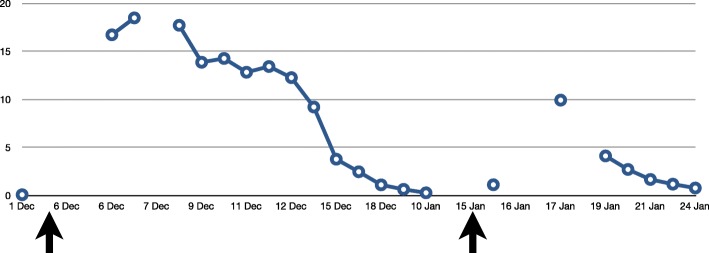
Trend of C-reactive protein (CRP) before and after first- and second-stage surgeries (arrows)

On postoperative day 1, laboratory tests showed a second, even more significant, increase in the amylase (569 U/L) and lipase level (2133 U/L) and a moderate increase in the CRP (9.93 mg/dL). Patient complained of mild pain at pressure in the abdomen. First defecation occurred on postoperative day 3 and abdominal pain resolved without further issues. In the following days, pancreatic enzymes gradually normalized and the patient was transferred to the floor on postoperative day 4 and then home 9 days after the procedure. Therapy with somatostatin, rifaximin, and ursodehoxycholic acid was maintained until complete normalization of amylase and lipase. Trend of hepatic enzymes (AST, ALT, GGT), pancreatic enzymes, and C-reactive protein before and after first- and second-stage surgeries is represented in Figs. 4, 5, and 6.

## Discussion and conclusions

As previously reported in literature [3, 7, 8], acute pancreatitis can occur after spine surgery, severely complicating the postoperative course for the patient. A prolonged number of fasting days and longer hospitalization are in fact to be expected in patients with postoperative pancreatitis [9].

Incidence varies among previous studies between 0.2 [10] and 7.4% [1]. Children with cerebral palsy undergoing spine surgery for scoliosis are the most likely to develop postoperative acute pancreatitis after posterior spinal fusion, with an incidence up to 30–55% [10, 11]. Correlation with lower body mass index (BMI), severe bleeding, intraoperative hypotension, preoperative Cobb angle of the main curve, and correction rate has been reported [10]. In contrast with other literature, Laplaza et al. found only older age and lower body index mass to be related to postoperative pancreatitis in 80 adolescents undergoing surgery for idiopathic scoliosis, thus excluding intraoperative bleeding and hypotension among risk factors [3]. Nevertheless, the hypothesized pathogenic mechanism is somehow related to hypoperfusion of the pancreas, caused by intraoperative hemodynamic instability or aggressive intraoperative hypotensive regimens to minimize blood losses, together with prolonged prone position which reduces retroperitoneal perfusion, thus leading to pancreatic ischemia [10]. Another advocated etiology is represented by mechanical compression of the pancreas, which increases with the amount of surgical correction of scoliosis. It is however still debated whether longer segments of fusion, the inclusion of the first lumbar vertebrae L1-L2, and the degree of correction may be independent risk factors for acute pancreatitis following scoliosis surgery [3, 10], while a correlation between lower BMI and the incidence of this complication seems accepted throughout literature. Less retroperitoneal fat may in fact easily expose the pancreas to direct compression against the vertebral column as described for blunt pancreatic traumatic injuries which are in fact more common in children and young adults because of a thinner or absent layer of protective adipose tissue around the pancreas [5].

Acute pancreatitis (AP) is characterized by the autolysis of the pancreas. Trypsinogen is converted to trypsin causing inflammatory changes and leading to benign pancreatic edema or to pancreatic or peripancreatic necrosis, with poorer prognosis. The Atlanta modified guidelines have been redacted for pancreatitis diagnosis in adults [4]. In 2012, the INSPPIRE (International Study Group of Paediatric Pancreatitis: In search of a cuRE) criteria were published to guide diagnosis of acute pancreatitis in children. According to these criteria, acute pancreatitis can be recognized when there are two of the following: clinical symptoms (abdominal pain of acute onset, nausea, vomiting, and back pain) and increased levels of serum amylase and/or lipase at least three times greater than the upper limit of normal and imaging finding characteristic [12]. As a result of inflammation, a recent retrospective review of 76 cases of acute pancreatitis in patients of 1–12 years of age found increased C-reactive protein level in 38% and leukocytosis in 33% of children. Diagnosis is mainly clinical [13]. Ultrasonography has a high rate of false negative, especially in the early phase or in mild cases [14]. Nevertheless, some typical findings in ultrasound examination may help the diagnosis of acute pancreatitis, including swelling, changes in echogenicity of the pancreas, or fluid collections. When suspected, a computed tomography (CT) scan of the abdomen may support the clinical diagnosis with findings of changes specific to pancreatitis [15]. According to the INSPPIRE group, if there are two criteria of acute pancreatitis, there is no indication to perform CT of the pancreas [4] due to low sensitivity (47–81%) [16], especially in the first phase of pancreatitis (edematous). A CT scan is instead recommended when necrosis is suspected [17] or to exclude hemorrhage in this context [10].

When acute pancreatitis is suspected, consultation with a gastroenterologist is suggested [10] for both diagnosis confirmation and intervention. Fasting and abdominal decompression with nasogastric tube are suggested. Total parenteral nutrition could support feeding during the early phase. Pharmacological interventions include proton-pump inhibitors and somatostatin intravenously. Patients must be monitored accurately for vital signs and abdominal objectivity. Laboratory tests are required to follow up pancreatic and liver enzymes until normalization.

Our case report presents some specific features. The patient presented only some of the risk factors associated with postoperative acute pancreatitis: she was proposed for high-grade correction surgery and long segments of fusion but in two stages, and her BMI was normal. The patient had conspicuous intraoperative blood losses and underwent massive transfusion.

The postoperative presentation of the complication allowed prompt diagnosis because two out of three INSPPIRE criteria were met on postoperative day 1 (mild abdominal pain during pressure, nausea and vomiting, elevated amylase, and lipase levels). Differential diagnosis with superior mesenteric artery syndrome (SMAS) [18] had to be clarified, and confirmation was obtained on postoperative day 2 with a CT scan showing abundant free fluid and no constriction of the duodenum.

To avoid aggressive immediate correction of scoliosis, patients with higher grade of deformity undergo two-stage posterior arthrodesis at our institution, including first-stage instrumentation with a growing magnetic bar and second-stage posterior fusion [19]. Although we waited for complete normalization of laboratory tests before scheduling the second stage procedure, the patient presented another elevation of pancreatic enzymes postoperatively after second surgery. The second procedure lasted 3 h and did not require any blood transfusion. Nevertheless, on postoperative day 1, the patient started complaining of abdominal pain during pressure and nausea and showed a greater than threefold increase in pancreatic enzymes in laboratory tests. The recurrence of the complication may testify a subjective predisposition to the complication after prone position surgery and stretching of the retroperitoneum or a partial clinical resolution of the first episode although laboratory tests normalized completely preoperatively and preoperative ultrasound scan excluded a residual pancreatic edema.

The gastroenterologist who evaluated the patient after the first surgical procedure described the ultrasound evidence of cholecystic sludge and referred sphincter contraction related to morphine administration as another possible etiology. The hypothesis of a cholecystic origin for the pancreatitis may be considered, especially when liver enzymes are elevated. Opioid consumption should be reduced to the minimum in cases of postoperative pancreatitis in scoliosis surgery in adolescents.

The benign evolution of the case here presented is consistent with prognosis of postoperative pancreatitis in this population in previous literature.

In conclusion, early recognition of symptoms (e.g., abdominal pain, tenderness, abdominal distension, nausea/vomiting, hypoactive bowel sounds, anorexia) plays a key role in preventing severe morbidity after scoliosis surgery. When symptoms suggest abdominal complication, pancreatic and liver enzymes are to be evaluated for posing prompt diagnosis. Gastroenterologic consultation and eventual imaging are further steps in differential diagnosis and treatment of this rare complication.

## Additional files

Additional file 1: Three-dimensional CT scan of the column. The image shows a 3D CT scan of the patient’s column. (JPG 316 kb)

Additional file 2: Column X-rays. The image shows the 70° kyphosis. (JPG 205 kb)

## References

[1] Feng F, Tan H, Li X, Qiao Y, Chen C, Lin Y, Li Z, Shen J. Incidence and risk factors of acute pancreatitis following scoliosis surgery: a prospective study. Spine. 2017; [Epub ahead of print]. 10.1097/BRS.0000000000002389 29016446

[2] Leichtner AM, Banta JV, Etienne N (1991). Pancreatitis following scoliosis surgery in children and young adults. J Pediatr Orthop.

[3] Laplaza FJ, Widmann RF, Fealy S (2002). Pancreatitis after surgery in adolescent idiopathic scoliosis: incidence and risk factors. J Pediatr Orthop.

[4] Banks PA, Bollen TL, Dervenis C (2013). Classification of acute pancreatitis--2012: revision of the Atlanta classification and definitions by international consensus. Gut.

[5] Debi U, Kaur R, Prasad KK (2013). Pancreatic trauma: a concise review. World J Gastroenterol.

[6] Choi E, Yaszay B, Mundis G, Hosseini P, Pawelek J, Alanay A, Berk H, Cheung K, Demirkiran G, Ferguson J, Greggi T, Helenius I, La Rosa G, Senkoylu A, Akbarnia BA (2017). Implant complications after magnetically controlled growing rods for early onset scoliosis: a multicenter retrospective review. J Pediatr Orthop.

[7] Thompson JS, Bragg LE, Hodgson PE, Rikkers LF (1988). Postoperative pancreatitis. Surg Gynecol Obstet.

[8] Rajaraman V, Heary RF, Livingston DH (2000). Acute pancreatitis complicating anterior lumbar interbody fusion. Eur Spine J.

[9] Borkhuu B, Nagaraju D, Miller F, Moamed Ali MH, Pressel D, Adelizzi-Delany J, Miccolis M, Dabney K, Holmes L (2009). Prevalence and risk factors in postoperative pancreatitis after spine fusion in patients with cerebral palsy. J Pediatr Orthop.

[10] Feng F, Tan H, Li X, Qiao Y, Chen C, Lin Y, Li Z, Shen J (2018). Incidence and risk factors of acute pancreatitis after scoliosis surgery: a prospective study. Spine.

[11] Abousamra O, Nishnianidze T, Rogers KJ (2018). Risk factors for pancreatitis after posterior spinal fusion in children with cerebral palsy. J Pediatr Orthop B.

[12] Morinville VD, Husain SZ, Bai H (2012). Definitions of pediatric pancreatitis and survey of present clinical practices. J Pediatr Gastroenterol Nutr.

[13] El Bouyousfi M, Leveque C, Miladi L (2016). Acute pancreatitis following scoliosis surgery: description and clinical course in 14 adolescents. Eur Spine J.

[14] Restrepo R, Hagerott HE, Kulkarni S (2016). Acute pancreatitis in pediatric patients: demographics, etiology, and diagnostic imaging. AJR Am J Roentgenol.

[15] Grzybowska-Chlebowczyk U, Jasielska M, Flak-Wancerz A, Więcek S, Gruszczyńska K, Chlebowczyk W, Woś H (2018). Acute pancreatitis in children. Gastroenterology Rev.

[16] Park A, Latif SU, Shah AU (2009). Changing referral trends of acute pancreatitis in children: a 12-year single-center analysis. J Pediatr Gastroenterol Nutr.

[17] Bai HX, Lowe ME, Husain SZ (2011). What have we learned about acute pancreatitis in children?. J Pediatr Gastroenterol Nutr.

[18] Altiok H, Lubicky JP, DeWald CJ (2005). The superior mesenteric artery syndrome in patients with spinal deformity. Spine.

[19] Greggi T, Maredi E, Vommaro F, Lolli F, Martikos K, Giacomini S, Di Silvestre M, Baioni A, Scarale A, Morigi A, Bacchin MR (2016). Innovative method of gradual temporary distraction using magnetic growing rods (MCGR) for surgical treatment of severe kyphoscoliosis: mini-case series. J Spine.

