# Supplementary figures and images for: Acute pancreatitis after major spine surgery: a case report and literature review
Source: Scoliosis Spinal Disord. 2018 Nov 8;13:24. doi: 10.1186/s13013-018-0170-2 (PMC6222983; doi:10.1186/s13013-018-0170-2)

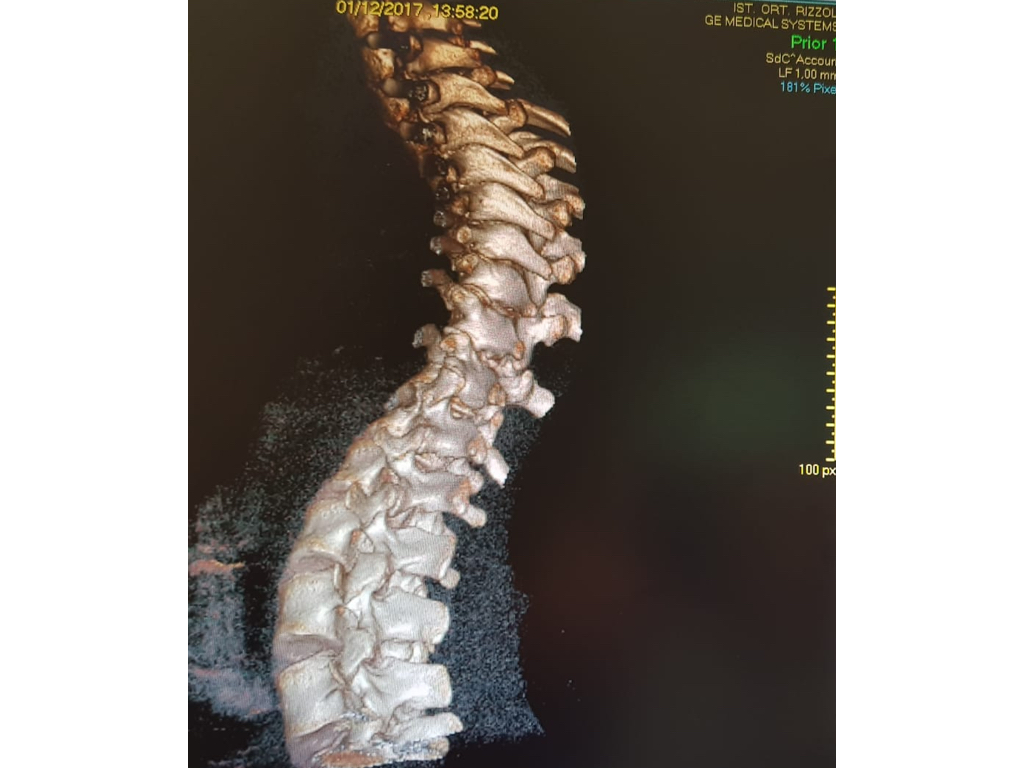

Supplement: Supplementary file 1 — Three-dimensional CT scan of the column. The image shows a 3D CT scan of the patient’s column. (JPG 316 kb) [file 13013_2018_170_MOESM1_ESM.jpg]

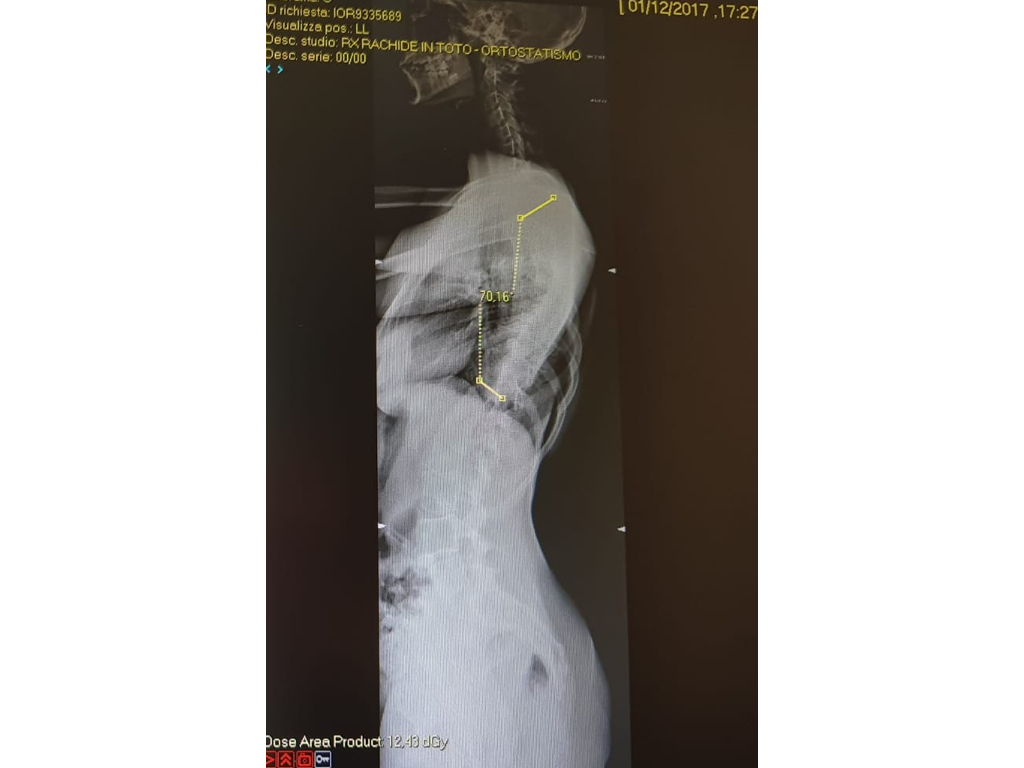

Supplement: Supplementary file 2 — Column X-rays. The image shows the 70° kyphosis. (JPG 205 kb) [file 13013_2018_170_MOESM2_ESM.jpg]
